# The effects of the *PHF6* gene mutation on myeloid neoplasms. A single-center cohort underpinned by a systematic review of literature

**DOI:** 10.1007/s00277-026-06766-y

**Published:** 2026-02-04

**Authors:** Edwin U. Suárez, Carlos J. Atencia, Fabio A. Torres-Saavedra, Nazareth Conejero, Rocío Salgado, Mireia Atance-Pararisas, Sara Perlado, Carlos Soto, Juan M. Alonso-Domínguez, Teresa Arquero-Portero, Raquel Mata, Elena Jiménez, J. L. López-Lorenzo, Álvaro V. Arriero, Juana Serrano-López, Socorro M. Rodríguez-Pinilla, Pilar Llamas

**Affiliations:** 1 Hematology Department, Fundación Jiménez Díaz University Hospital, Universidad Autónoma de Madrid, Madrid, Spain; 2 Pathology Department, Fundación Jiménez Díaz University Hospital, Madrid, Spain; 3 Internal Medicine Department, Universidad de Antioquia, Medellín, Colombia; 4 Experimental Hematology Lab, IIS-Fundación Jiménez Díaz, Universidad Autónoma de Madrid, Madrid, Spain

**Keywords:** Acute myeloid leukemia, Myelodisplastic syndrome, Myelodysplastic-myeloproliferative diseases, Myeloproliferative disorders, *PHF6*

## Abstract

The mutation of the plant homeodomain finger protein 6 gene (*PHF6*^*MUT*^) in patients with myeloid neoplasms (MNs) is rare and appears to play a role in prognosis, though this is still under debate. We conducted a retrospective analysis of a cohort of 313 patients diagnosed with MNs. We also performed a systematic review (SR) of the literature to evaluate the prognostic role of *PHF6* gene status in MNs. We identified 15 patients with *PHF6*^*MUT*^. In the multivariate analysis, *PHF6*^*MUT*^ was associated with higher mortality compared to *PHF6*^*wild − type*^ (hazard ratio [HR] = 1.02; 95% confidence interval [CI], 1.00–1.05; *P* = 0.075), with no apparent impact from other co-mutations. In the multilevel logistic model by MN subtype, the presence of *PHF6*^*MUT*^ (independent of variant allele frequency > 20%) was shown to have a positive coefficient (adverse prognosis) in acute myeloid leukemia; in the remainder of MNs, the effect was not significant. *PHF6*^*MUT*^ had a marginal and significant effect compared to *PHF6*^*wild − type*^ cases (HR = 1.02; 95% CI, 1.00-1.05; *P* = 0.039). There were no significant differences in time to blast transformation or time to next treatment depending on *PHF6* gene status. According to the results of most studies published to date (SR), *PHF6*^*MUT*^ has a prognostic role in MNs; our results are consistent in terms of clinical outcomes, but these marginal effects should be interpreted with caution in the context of existing prognostic models given the limitations of the small sample size.

**Supplementary Information:**

The online version contains supplementary material available at 10.1007/s00277-026-06766-y.

Some somatic mutations of *PHF6* (*PHF6*^*MUT*^) have been identified in myeloid neoplasms (MNs), although at a low frequency (3.1%) [1, 2]. *PHF6*^*MUT*^ is currently included in the risk stratification of myelodysplastic syndromes (MDS), and its presence is associated with an unfavorable prognosis [3]. In chronic myelomonocytic leukemia (CMML), however, more recent data suggest a favorable prognosis [2, 4, 5]. In other MNs, the situation is less clear, with heterogeneous data [2, 6–8].

A total of 313 patients diagnosed with MNs were identified at our center from 2017 until 2025 (January). The mean age at diagnosis was 71 years (interquartile range [IQR] 60–80), and 43% (*n* = 144) were men. There were 51 deaths (16%), mainly in the acute myeloid leukemia (AML [46%]) and MDS (23%) subgroups. The mean follow-up was 440 days (IQR 209–818). The mean overall survival (OS) was 157 days (IQR 51–518) (Table 1). All the patients underwent evaluation to obtain an integrated diagnosis of MN. This included next-generation sequencing (NGS), which contained the *PHF6* gene in its panel (Supplementary appendix; p. 1). Details of the ethics statement and statistical methodology can be found in the supplementary appendix (p. 1–2).

**Table 1:** General characteristics of the cohort according to each myeloid neoplasm

Characteristic	MNs, *N* = 313^*1*^	AML, *N* = 47^*1*^	MDS, *N* = 78^*1*^	MDS/MPN, *N* = 50^*1*^	MPN, *N* = 138^*1*^	*p*-value^2^
Age, years (IQR)	71 (60–80)	67 (52–78)	77 (69–85)	75 (66–82)	66 (54–76)	< 0.001
Females; N(%)	144 (46)	17 (35)	33 (42)	17 (34)	77 (56)	0.015
WBC (x10^9^/L)	7.5 (4.9–10.8)	13.3 (2.3–32.7)	4.3 (2.9 − 5.9)	9.8 (6.7 − 17.6)	8.5 (6.5–10.7)	< 0.001
Hb (gr/dL)	12 (9.7–14.5)	9 (7.6–10)	10.2 (8.9–11.6)	11.9 (10.2–13.1)	14.7 (13.0–16.3)	< 0.001
Blasts PB; % (range)	0 (0–0)	43 (17–73)	0 (0–0)	0 (0–0)	0 (0–0)	< 0.001
N x10^9^/L (IQR)	3.8 (2.1–6.4)	1.4 (0.3–3.6)	2.0 (1.2–3.2)	6.4 (3.4–10.1)	5.2 (3.7–7.3)	< 0.001
L x10^9^/L (IQR)	1.8 (1.3 − 2.6)	1.7 (1.2–4.0)	1.5 (1.1–2.1)	2 (1.3 − 2.8)	2.1 (1.5 − 2.6)	0.001
M x10^9^/L (IQR)	0.6 (0.4 − 1.0)	0.9 (0.2–2.3)	0.4 (0.3 − 0.7)	1.2 (0.7 − 2.2)	0.6 (0.5 − 0.8)	< 0.001
Platelets x10^9^/L (IQR)	241 (82.5–550)	62.5 (32–95)	100 (60–222)	189.5 (85–309)	573.5 (405–714)	< 0.001
Thrombocytopenia						< 0.001
<150 × 10^9^/L (%)	120 (38)	43 (91)	51 (65)	21 (42)	5 (3.6)	
≥150 × 10^9^/L (%)	193 (62)	4 (8.5)	27 (35)	29 (58)	133 (96)	
*PHF6*^*MUT*^; N (%)	15 (4.8)	3 (6.4)	6 (7.7)	4 (8.0)	2 (1.4)	0.047
*Missense*	8 (2.6)	2 (4.3)	3 (3.8)	2 (4)	1 (0.7)	
*Nonsense*	3 (1.0)	1 (2.1)	1 (1.3)	1 (2)	0 (0)	
*Frameshift*	4 (1.3)	0 (0)	2 (2.6)	1 (2)	1 (0.7)	
*VUS*	5 (1.6)	2 (4.3)	1 (1.3)	2 (4)	0 (0)	
*P/LP*	10 (3.2)	1 (2.1)	5 (6.4)	2 (4)	2 (1.4)	
Normal Karyotype; N (%)	208 (66)	18 (38)	42 (54)	40 (80)	108 (78)	
Abnormal Karyotype; N (%)	90 (29)	25 (53)	28 (36)	7 (14)	30 (22)	
Complex Karyotype; N (%)	12 (3.8)	3 (6.4)	7 (9.0)	2 (4.0)	0 (0)	
Cytog. Abnorm. chr.7	9 (2.9)	3 (6.4)	4 (5.1)	2 (4.0)	0 (0)	
Cytog. Abnorm. 17p/*TP53*	6 (1.9)	1 (2.1)	3 (3.8)	2 (4.0)	0 (0)	
Deaths; N (%)	51 (16)	21 (46)	18 (23)	7 (14)	5 (3.6)	< 0.001
AlloHCT; N(%)	19 (6.1)	9 (19)	7 (9.1)	1 (2.0)	2 (1.5)	< 0.001
Survival, days; median (IQR)	157 (51–518)	86 (39–202)	299 (109–497)	375 (57–1013)	1251 (783–2155)	0.009
TTBT, months; median (IQR)	521 (426–1695)	N.A.	441 (426–491)	786 (407–1165)	1901 (1129–2205)	0.045
T2L, months (%)	48 (15)	7 (15)	16 (21)	9 (18)	16 (12)	0.3

^*1*^Median (Q1 - Q3); n (%)

^*2*^Kruskal-Wallis rank sum test; Pearson’s Chi-squared test; Fisher’s exact test

*AML* acute myeloid leukemia, *MDS* Myelodisplastic Syndrome, *MPN* Myeloprolipherative Neoplasm, *MDS/MPN* Overlap MDS and MPN, *IQR* Interquartile Range, *WBC* White Blood Count, *Hb* Hemoglobin, *PB* Peripheral Blood, *N* Neutrophil Count, *L* Lymphocyte count, *M* Monocyte count, *VUS* Variants of Uncertain Significance, *P/LP* Patogenic/Likely Patogenic, *Cytog. Abnorm.* Cytogenetic Abnormalities, *Cr.* Chromosome, *AlloHCT* Allogeneic Hematopoietic Cell Transplantation, *TTBT* Time-to-Blast Transformation, *T2L* Time-to-second line of treatment, *N.A.* Not Applicable

Fifteen patients with *PHF6*^*MUT*^ (4.8%) were found: six cases with MDS, four with MDS/Chronic Myeloproliferative Neoplasm overlap (MDS/MPN; specifically CMML), two with MPN, and three with AML. Five of these mutations had not been reported previously and were categorized as variants of uncertain significance (Table 1). The different *PHF6*^*MUT*^ were classified as missense (*N* = 8; 53.3%), frameshift (*N* = 4; 26.7%), and nonsense (*N* = 3; 20%). On average, the variant allele frequency (VAF) of *PHF6*^*MUT*^ was 26.1% (IQR 0.39–42.2). At least one co-mutation was expressed in all patients with *PHF6*^*MUT*^; the most frequent was *ASXL1* (*N* = 9; VAF average 30.6%) (Supplementary appendix. Figure S1).

Of the patients with *PHF6*^*MUT*^, 73% were male (*N* = 11), and the age of diagnosis was later than in *PHF6*^*wild − type*^ patients (Figure S1). Likewise, a higher VAF was observed in males than in females (Figure S1). The correlation of *PHF6*^*MUT*^ and its co-mutations was represented using a corrplot matrix in all MNs and by diagnostic subcategories (Figures S2 and S3). In the MN group, no positive or negative correlation was observed. In the MPN subcategory, there was a positive correlation with *ASXL1* and *STAG2*, and in AML with *FLT3* (within both, internal tandem duplication and tyrosine kinase domain).

In the Random Forest models, the Gini index was calculated to reflect the importance of each mutation in mortality; *PHF6*^*MUT*^ was found to have an adverse impact, with a lower amplitude than other mutations such as *TP53* (Figure S4). In the bivariate analysis, according to *PHF6* status and death, OS was not different (*P* = 0.44) (Figure S5). In the multivariate analysis, which included diagnostic subcategories according to The 5th World Health Organization classification (WHO-5), the presence of *PHF6*^*MUT*^ was found to harm OS (Hazard ratio [HR] = 1.02; 95% confidence interval [CI], 1.00–1.05; *P* = 0.075) (Table S2) [9]. When the co-mutational profile was included, a significant impact on mortality was observed, with an HR of 1.03 (95% CI, 1.00–1.06; *P* = 0.024) (Table S3). In the multilevel logistic model by MN subtype, *PHF6*^*MUT*^ appears to have an adverse effect on AML (*N* = 3; coefficient = 4.9), while its impact on MDS (*N* = 6; coefficient = 1.0), MDS/MPN (*N* = 4; coefficient = −1.1) and MPN (*N* = 2; coefficient = −1.9) remains uncertain, and does not appear to be dependent on clonal burden (Fig. 1).

**Fig. 1:**
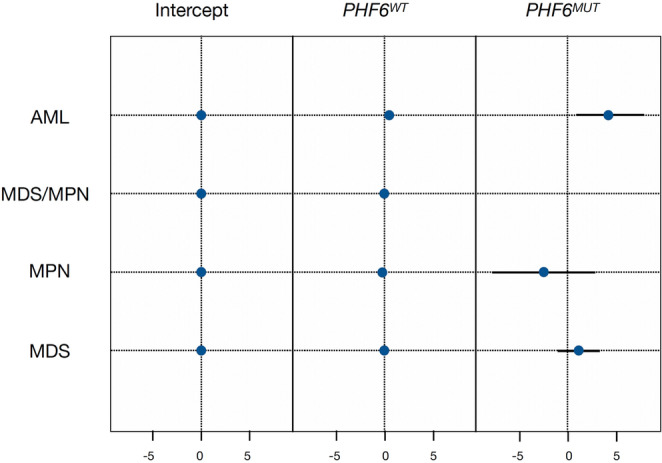
Overall survival in each myeloid neoplasm subgroup according to *PHF6* gene status. Effect expressed in log-odds. AML: Acute Myeloid Leukemia; MDS: Myelodiplastic Syndrome; MPN: Myeloproliferative Neoplasm; MDS/MPN: overlap MDS and MPN; *PHF*^*WT*^: *PHF6* Wild Type; *PH6*^*MUT*^: *PHF6* mutated

No differences were found in the bivariate or multivariate analyses concerning *PHF6*^*MUT*^ for time-to-next treatment (Tables S4 and S5) and time-to-blastic transformation (Tables S6 and S7). No significant differences were found concerning progression-free survival (PFS) according to *PHF6*^*MUT*^ status in the bivariate analysis (Figure S6), but in the multivariate analysis, it had a marginal effect of 2% (HR = 1.02; 95% CI, 1.00–1.05; *P* = 0.039) (Tables S8 and S9).

In line with other publications, we confirm a low incidence (4.8%) of *PHF6*^*MUT*^ in MNs [1, 8]. On average, the VAF of *PHF6*^*MUT*^ was low (26.1%; IQR 0.39–42.2), supporting the hypothesis that it is a late event in the evolution of MNs [1, 8].

The co-mutation pattern suggests that *PHF6*^*MUT*^ is not an isolated event, but part of shared mutational changes. Regardless of the MN subtype, the most frequent co-mutations were *ASXL1*,* TET2*, and *RUNX1*, consistent with previous work on MNs [5, 8, 10]. (Figure S1). In our study, no clear correlation with other mutations is seen in the overall analysis of MNs (Figure S2). In the analysis by MN subtype, *PHF6*^*MUT*^ appears to show a greater correlation with *ASXL1* and *STAG2* mutations only in MPN (*N* = 2; chronic myeloid leukemia and primary myelofibrosis) and in AML (*N* = 3) with *FLT3* (Figure S3).

Previous studies have found differences in the co-mutation profile depending on sex [6]. A higher number of higher-risk or adverse prognosis co-mutations (*TP53*,* ASXL1*,* RUNX1*) was also observed in the male population (*N* = 11). These factors could amplify the clinical effect of *PHF6*^*MUT*^ [7].

A systematic review (SR) of the literature was conducted to evaluate the prognostic role of *PHF6*^*MUT*^ in MNs. The details of the search are described in the supplementary appendix. The literature analysis flowchart is shown in Figure S7. Based on the results, we can infer that there is a frequent association between poor prognosis and *PHF6*^*MUT*^. The studies included in this SR are shown on the colour map, with a greater number of results with an adverse effect in most MNs (Table S1). This association is often more evident in combination with other mutations such as *RUNX1*,* ASXL1*, or *TP53*. Studies with a favorable effect were less frequent and correspond to some studies where *PHF6*^*MUT*^ could better predict response to antineoplastic treatments. More recent studies that identified favourable prognoses focused on CMML [2, 4].

However, given the nature of the data found in this SR (low mutation rate, retrospective design, statistical tools, heterogeneity of the different MNs, effect of co-mutations, etc.), it may not be accurate or universal to conclude a specific effect in each MN.

*PHF6*^*MUT*^ is associated with worse outcomes although marginal in terms of OS and PFS when additional molecular and cytogenetic characteristics are included in the multivariate model (Tables S3 and S9); furthermore, it can be observed that the other co-mutations have no impact on these outcomes. In the multilevel analysis, it was observed that *PHF6*^*MUT*^ exerts an adverse prognostic effect in AML but not in other hematological malignancies (Fig. 1). These minimal effects should be interpreted in the context of existing prognostic models with caution. A new risk model for CMML (BLAST-mol), which incorporates molecular information to help identify low- or intermediate-risk patients, was published more recently [5]. This score considers *PHF6* and *TET2* mutations to be favourable, indicating a more indolent course for patients categorised as intermediate risk [5].

Our results are consistent in terms of clinical outcomes across different diagnostic categories where *PHF6* appears to indicate a poor prognosis; however, we cannot determine the effect of *PHF6* in the CMML subgroup or MPN. Due to the small sample size, subgroup analysis of myeloid neoplasms reduces the statistical power and reliability of the results (potential overfitting and spurious associations). It is necessary to evaluate whether, by expanding the number of patients and centers, the results are different when defining the clinical/prognostic impact of *PHF6*^*MUT*^.

## Supplementary Information

Below is the link to the electronic supplementary material.

Supplementary Material 1 

## Data Availability

Data is available upon request.
