# Supplementary material for: The effects of the PHF6 gene mutation on myeloid neoplasms. A single-center cohort underpinned by a systematic review of literature
Source: Ann Hematol. 2026 Feb 4;105(3):77. doi: 10.1007/s00277-026-06766-y (PMC12868061; doi:10.1007/s00277-026-06766-y)
Supplement: Supplementary file 1 — Supplementary Material 1 [file 277_2026_6766_MOESM1_ESM.docx]

**Supplementary appendix.**

**Methods.**

**The next generation sequencing (NGS) panels.** We used two NGS panels due to an update to the test in 2022. Both included the change to the *PHF6* gene.

(1) Panmyeloid Consortium Panel (Panmyeloid_v1) from SophiaGenetics, which includes the genes mentioned in Appendix 1; sequencer: Illumina NextSeq; minimum depth: 1000x; analysis software: Sophia DDM; databases consulted: COSMIC, ExAC, 1000Genomes, ClinVar, P53 Database.

(2) Haematology OncoKitDx CE IVD, Healthincode, Imegen. Ref: IMG-363. The genes included in the panel are listed in Appendix 1; sequencer: Illumina NextSeq; minimum depth: 200x; analysis software: DataGenomics (Imegen); databases consulted: COSMIC, ExAC, 1000Genomes, ClinVar, P53 Database, Varsome.

**Statistical methods.**

Some variables were scaled for random forest models. Missing data (<5%) was evaluated, and a multiple imputation procedure was performed using the K-nearest neighbors method. Descriptive statistical procedures were used, employing the median and interquartile range (IQR) for quantitative variables and percentage and absolute number for qualitative variables. To estimate the difference between them, a Mann-Whitney U test and a chi-square test were used, respectively, with a significance level of *P* < 0.05. For the bivariate Pearson correlation analysis, the quantitative variables of percentage of variant allele frequency (%VAF) were taken for each *PHF6^MUT^* and other genetic mutations. Three multivariable Cox regression models were adjusted for time to death (OS), use of second-line treatment (time-to-next therapy [TTNT]), blast transformation (time to blast transformation [TTBT]), and progression-free survival (PFS). In addition, considering the number of *PHF6^MUT^*, the Firth-type Cox models were performed. Assumptions of proportional hazards, log-hazard linearity, and non-collinearity were validated. The C statistic and other performance metrics were calculated. The hazard ratio (HR) and its respective 95% confidence interval (95% CI) were used to estimate the effect. Survival curves were used to visualize the time-to-event outcomes, and the probability of death was calculated using the Kaplan-Meier method. The median survival was estimated for each group of myeloid neoplasms. In addition, considering possible nonlinear effects, models were adjusted with restricted cubic splines with 4 degrees of freedom and fractional polynomials for variables such as % variant allele frequency (VAF) of *PHF6^MUT^*, peripheral blood blast count, and age. Finally, given the potential variation in the prognostic significance of *PHF6^MUT^* reported in the literature across different myeloid neoplasms (MNs), the number of *PHF6^MUT^* individuals, and the number of OS outcomes, a multilevel logistic model was adjusted using the diagnosis category (5^th^ World Health Organization Classification [WHO-5]) variable to estimate the random effects of exposure to *PHF6^MUT^.* To select model characteristics and evaluate the robustness of the findings, RF models were used, with 500 trees and 1000 interactions and an average of 6 nodes using the VAF variables of switched genes. The Gini index was calculated as a measure of total variance and a measure of “node” (bifurcation) purity. Statistical analyses were performed using the specialized statistical software R and its interface, RStudio version 4.2.2 (R Core Team (2023). R: A Language and Environment for Statistical Computing. R Foundation for Statistical Computing, Vienna, Austria. <[https://www.R-project.org/](https://www.r-project.org/)>).

**Ethical statement.**

The study was conducted by the recommendations contained in the Declaration of Helsinki (5th WMA General Assembly, Helsinki, Finland, October 2024; World Medical Association. World Medical Association Declaration of Helsinki: Ethical Principles for Medical Research Involving Human Participants. *JAMA*. 2025;333(1):71-74. doi:10.1001/jama.2024.21972), and the Standards of Good Clinical Practice.

**Systematic review methodology.**

A systematic review of the literature was conducted, following the recommendations of Preferred Reporting Items of Systematic Reviews and Meta-Analyses (PRISMA) to evaluate the prognostic role of *PHF6^MUT^* in MNs.. The search was conducted in MEDLINE, EMBASE, and Cochrane Library, using the terms “*PHF6*” and “mutation” (the following limits were used: language [English, Spanish, French]; Abstract, Full text, Humans). Studies that had evaluated the prognostic value (OS, PFS, response to treatment, blast transformation, etc.) of *PHF6^MUT^* exclusively in MNs were selected. Bibliographic references from the most representative studies and reviews on the subject were also evaluated. Unpublished works in these databases were searched through the supplementary volumes of the most relevant conferences in the fields of hematology and hemopathology.

**Results**

**Tables.**

**Table S1. Studies that consider the role of *PHF6^MUT^* as a prognostic factor.**

| **Study* / Date** | **N of patients/MN** | **Main results** | **Study design** |
| --- | --- | --- | --- |
| *Patel*  *(ECOG E1900 trial; USA),* 2012 | 398; AML | *PHF6^MUT^* 3%. Univariate analysis: Associated with reduced OS; identifies the intermediate risk group. Not significant in multivariate analysis. | Prospective; *post hoc.* |
| *Thota (USA),* 2013 | 168; MDS | Among the mutations that predict response, *PHF6* (OR 4.22). | Retrospective; Meeting Abstract. |
| *Heuser* (Germany), 2015 | 308; MDS or secondary AML | *PHF6^MUT^* 4.9%; impact on OS (HR 2.2). In addition, mutations in *PTPN11* and *PHF6* predicted a high incidence of non-relapse mortality. | Retrospective; Meeting Abstract. |
| *Mori* (Japan), 2016. | 1760; MNs | *PHF6^MUT^* 3.1%. There was no association with prognosis (OS). | Retrospective. |
| *Gaidzik* (Germany), 2016. | 2439; AML | *PHF6^MUT^ (3%). PHF6^MUT^/ RUNX1^MUT^had lower responses than PHF6^wild-type^/RUNX1^MUT^* | Retrospective. |
| *Sloan* (*USA*), 2016. | 197; AML | *PHF6^MUT^* 2.5 %. In the IGP model, patients with *FLT-ITD^Wild Type^* with high-risk co-mutations (*TET2, ASXL1,* and/or *PHF6*); “unfavorable”) had a similar OS to patients with “intermediate risk.” | Retrospective. |
| *Lee (USA)*, 2016. | 94; MDS | *PHF6^MUT^* n= 5. The gain of mutations in *CBL, MPL, NPM1*, or *PHF6* was associated with poor OS (HR 8.15, *P*=0.0008, CI, 2.38-27.86). | Retrospective; Meeting Abstract. |
| *Yoshizato* (Japan), 2016 | 865; transplanted MDS | In the multivariate analysis, these mutations were associated with worse OS: *CBL* (HR: 2.72), *KRAS* (2.60), *EZH2* (2.37), *PHF6* (2.29), and *TP53* (1.95). | Retrospective; Meeting Abstract. |
| *Lin PH* (Taiwan), 2017. | 112; AML | *PHF6^MUT^* 2.7%. It was associated with worse OS (HR: 6,01 (1.25–28.84 CI 95%, *P*=0.025). | Retrospective. |
| *Madanat (USA),* 2017. | 527; MDS | *PHF6^MUT^ (P*=0.018*).* In the univariate analysis, it was associated with progression to AML. In the multivariate analysis, having ≥3 mutations (HR: 1.65; 95% CI, 1.1-2.5; *P*=0.021). | Retrospective; Meeting Abstract. |
| *Xiao (USA),* 2017 | 1329; MNs | *PHF6^MUT^* n=33 (2.5%). The 2-year OS for *PHF6^MUT^* in MPAL was lower compared to non-mutated cases (40% *vs*. 100% in *PHF6^wild-type^* [*P*=0.005]). | Retrospective; Meeting Abstract. |
| *Shaver (USA),* 2017. | 100; AML | A subgroup of genes associated with transcription (*ETV6, NMP1, PHF6, WT1*) was associated with high blast disease burden (cut-off point ≥20%). | Retrospective; Meeting Abstract. |
| *Lin Y* (China), 2020. | 156; AML | *PHF6^MUT^ 3.8%.* When *PHF6^MUT^* is accompanied by mutations in *ASXL1*, the prognosis for AML is not significantly affected. | Retrospective. |
| *Lachowiez (USA),* 2020. | 62; AML | Mutations in tumor suppressor genes (*TP53, WT1, PHF6*) were more frequent in non-responders (66% *vs.* 19%, *P*=0.014). | Prospective; *Interim* analysis 1b/2 clinical trial; Meeting Abstract. |
| *Jain (USA),* 2021. | 1914; MDS | *PHF6^MUT^* was associated with progression to HR-MDS and then to AML*(P=*0.0093). | Retrospective; Meeting Abstract. |
| *Huang* (China), 2022. | 801; AML | *PHF6^MUT^* 2.75%. Decrease in complete remissions, low response to induction chemotherapy, and lower OS. | Retrospective. |
| *Zhang* (China), 2022. | 176; CML with TK3G inhibitors. | *PHF6^MUT^* 9.5%. In the multivariate analysis, *ASXL1^G646Wfs*12^* and *PHF6^MUT^* were significantly associated with adverse cytogenetics and molecular response. Mutations in *RUNX1* and *PHF6* were associated with worse PFS. | Retrospective; Meeting Abstract.. |
| *Mahsoub (USA),* 2022. | 185; AML *de novo: 8* con *NF1^MUT^* | *PHF6^MUT^* n= 1/8. *NF1^MUT^* is uncommon in AML (4.3%) and is characterized by co-mutation with *PHF6* and poor prognosis *vs.* AML with normal karyotype. | Retrospective; Meeting Abstract. |
| *Zhou (USA),* 2022. | 62; *JAK2* negative MPN | Mutations in *TP53, PHF6,* or *RUNX1* are associated with a high risk of secondary AML. | Retrospective; Meeting Abstract. |
| *Bernard,* 2022. | 2957; MDS | *PHF6^MUT^* N=98 (3.31%). Included along with 14 other genes in a category of “residual genes” with an additive effect on adverse prognosis in the IPSS-Mol. | Retrospective. |
| *Zhao* (Canada), 2023. | 266; AML-MR | *PHF6^MUT^* (4,5%) and transplantation were predictors of better OS (HR 0.15 [0.03–0.87 95% CI] *P*=0.034) and DFS (0.15 [0.03–0.78 95% CI] *P*=0.024). | Retrospective. |
| *Bataller (USA),* 2023. | 1699; MNs | *PHF6^MUT^* 3.3%. No association with prognosis (OS). However, in the AML-TR subgroup, there was an increase in mortality in the multivariate analysis. | Retrospective. |
| *Tefferi (USA)*, 2024. | 426; CMML | *PHF6^MUT^* 6.6%. Better prognosis (OS), thrombocytopenia, and more *LoY.* | Retrospective. |
| *Erdos (USA),* 2024. | 475; PV | High mortality at 20 years, associated with advanced age (*P*<0.001) and *PHF6^MUT^* (*P*=0.013) in multivariate analysis. | Retrospective; Meeting Abstract. |
| *Beas* (Spain; GESMD)*,* 2024. | 209; MDS | *PHF6^MUT^* (4.3%), did not provide prognostic value in the univariate analysis, regardless of the event considered (PFS to AML or OS). | Retrospective; Meeting Abstract. |
| *Perusini* (Canada), 2024. | 92; Secondary AML | Tumor suppressors: *PHF6^MUT^* + *TP53^MUT^* (18.5%); this was the only subgroup that showed differences in OS (at 1 year, HR 3.11 [1.4-6.9], *P*<0.001) in favor of CPX *vs.* FLAG-IDA. | Retrospective; Meeting Abstract. |
| *Kubota (USA*, Italy and Germany*)*, 2025. | 8843; MNs | *PHF6^MUT^* 1.74%. Negative prognosis in AML (especially when associated with mutated *RUNX1*), as well as age >60 years. | Retrospective. |
| *Fathima (USA),* 2025**.** | 176; MNs | *PHF6^MUT^* N=176 (100%).  · Better prognosis in LMMC (OS and BTFS), higher LoY in CMML and MDS.  · Better prognosis (BTFS) in women with MDS.  · Better PTS. | Retrospective. |
|  |  | · Better prognosis (OS) in MDS with *PHF6^MUT^* VAF >20%. |  |

The colors in the **Main** **results** column mean the following:

**Green:** favorable prognosis

**Yellow:** neutral effect

**Red:** unfavorable prognosis

MN: Myeloid Neoplasm; AML: Acute Myeloid Leukemia; MDS: Myelodiplastic Syndrome; OR: Odds Ratio; OS: Overall Survival; HR: Hazard Ratio; IGP: Integrated Genetic Prognostic model (Patel et al. 2013); CI: Confidence Interval; MPAL: Mixed-Phenotype Acute Leukemia; HR-MDS: High-Risk MDS; CML: Chronic Myeloid Leukemia; TK3G: Tyrosine-Kinase 3rd. Generation; PFS; Progression-Free Survival; MPN: Chronic Myeloproliferative Neoplasm; IPSS-Mol: Molecular International Prognostic Scoring System for Myelodysplastic Syndromes*;* AML-MRC: AML with myelodysplasia-related changes; DFS: Disease-Free Survival; AML-TR: AML Therapy-Related; CMML: Chronic Myelomonocytic Leukemia; LoY: loss-of-Y chromosome; CPX: liposomal formulation of daunorubicin and cytarabine with a molar ratio of 1:5 [*Vyxeos*]); FLAG-IDA: Fludarabine, Leucovorin, Ara-C (cytarabine), G-CSF [granulocyte colony-stimulating factor], Idarubicin); PTS: Post-transplant Survival; BTFS: Blastic Transformation-Free Survival.

***Studies included:**

Patel JP, Gönen M, Figueroa ME, et al. Prognostic relevance of integrated genetic profiling in acute myeloid leukemia. *N Engl J Med*. 2012;366(12):1079-1089. doi:10.1056/NEJMoa1112304.

Thota S, Lakin P, Husseinzadeh H, et al. Somatic Mutational Screen For Improved Prediction Of The Outcomes Of Epigenetic Therapy In MDS. *Blood*. 2013;122(21):659. doi:[10.1182/blood.V122.21.659.659](https://doi.org/10.1182/blood.V122.21.659.659)

Heuser M, Koenecke C, Gabdoulline R, et al. Molecular Predictors of Outcome in Patients with MDS and AML Following MDS after Allogeneic Hematopoietic Stem Cell Transplantation. *Blood*. 2015;126(23):912. doi:[10.1182/blood.V126.23.912.912](https://doi.org/10.1182/blood.V126.23.912.912).

Mori T, Nagata Y, Makishima H, et al. Somatic PHF6 mutations in 1760 cases with various myeloid neoplasms. *Leukemia*. 2016;30(11):2270-2273. doi:10.1038/leu.2016.212.

Gaidzik VI, Teleanu V, Papaemmanuil E, et al. RUNX1 mutations in acute myeloid leukemia are associated with distinct clinico-pathologic and genetic features [published correction appears in Leukemia. 2016 Nov;30(11):2282. doi: 10.1038/leu.2016.207.]. *Leukemia*. 2016;30(11):2160-2168. doi:10.1038/leu.2016.126

Sloan CE, Luskin MR, Boccuti AM, et al. A Modified Integrated Genetic Model for Risk Prediction in Younger Patients with Acute Myeloid Leukemia. *PLoS One*. 2016;11(4):e0153016. Published 2016 Apr 6. doi:10.1371/journal.pone.0153016.

Lee S, Barnard J, DeZern AE, et al. Is Serial Monitoring of Myeloid Mutations Clinically Relevant in Myelodysplastic Syndromes (MDS): A Report on Behalf of the MDS Clinical Research Consortium (CRC). *Blood*. 2016;128(22):297. doi:[10.1182/blood.V128.22.297.297](https://doi.org/10.1182/blood.V128.22.297.297).

Yoshizato T, Shiozawa Y, Yoshida K, et al. Impact of Somatic Mutations on Outcome in Patients with MDS after Stem-Cell Transplantation. *Blood*. 2015;126(23):711. doi:[10.1182/blood.V126.23.711.711](https://doi.org/10.1182/blood.V126.23.711.711)

Lin PH, Li HY, Fan SC, et al. A targeted next-generation sequencing in the molecular risk stratification of adult acute myeloid leukemia: implications for clinical practice. *Cancer Med*. 2017;6(2):349-360. doi:10.1002/cam4.969.

Madanat YF, Sekeres MA, Al-Issa K, et al. Distinct Genomic Associations to Predict Acute Myeloid Leukemia (AML) Progression from Myelodysplastic Syndromes (MDS). *Blood*. 2017;130(Supplement 1):4245. doi:[10.1182/blood.V130.Suppl_1.4245.4245](https://doi.org/10.1182/blood.V130.Suppl_1.4245.4245).

Xiao W, Pastore F, Getta B, et al. PHF6 Mutations Defines a Subgroup of Mixed Phenotype of Acute Leukemia with Aberrant T-Cell Differentiation. *Blood*. 2017;130(Supplement 1):1384. doi:[10.1182/blood.V130.Suppl_1.1384.1384](https://doi.org/10.1182/blood.V130.Suppl_1.1384.1384)

Shaver AC, Juskevicius R, Daber RD, Strickland SA, Ferrell PB, Byrne M, et al. Mutational signature correlates with proliferative phenotype in NCCN poor-risk acute myeloid leukemia. Hematopathology. *Lab Invest* 97 (Suppl 1), 335–388 (2017). https://doi.org/10.1038/labinvest.2016.172

Lin Y, Wang Y, Zheng Y, Wang Z, Wang Y, Wang S. Clinical characteristics and prognostic study of adult acute myeloid leukemia patients with *ASXL1*mutations. *Hematology*. 2020;25(1):446-456. doi:10.1080/16078454.2020.1847801.

Lachowiez C, Konopleva M, Kadia TM, et al. Interim Analysis of the Phase 1b/2 Study of the BCL-2 Inhibitor Venetoclax in Combination with Standard Intensive AML Induction/Consolidation Therapy with FLAG-IDA in Patients with Newly Diagnosed or Relapsed/Refractory AML. *Blood*. 2020;136(Supplement 1):18-20. doi:[10.1182/blood-2020-134300](https://doi.org/10.1182/blood-2020-134300).

Jain AG, Ball S, Aguirre LE, et al. The Natural History of Lower Risk MDS: Factors Predicting Progression to High-Risk Myelodysplastic Syndrome and Acute Myeloid Leukemia in Patients with Very Low and Low Risk MDS According to the R-IPSS Criteria. *Blood*. 2021;138(Supplement 1):2600. doi:[10.1182/blood-2021-149708](https://doi.org/10.1182/blood-2021-149708).

Huang K, Wang L, Zheng Y, et al. PHF6 mutation is associated with poor outcome in acute myeloid leukaemia. *Cancer Med*. 2023;12(3):2795-2804. doi:10.1002/cam4.5173.

Zhang X, Li Z, Qin YZ, et al. Cancer-Related Gene Mutations Drive Resistance to the Third-Generation Tyrosine Kinase Inhibitor Therapy in Chronic Myeloid Leukaemia. *Blood*. 2022;140(Supplement 1):9611-9613. doi:[10.1182/blood-2022-169397](https://doi.org/10.1182/blood-2022-169397)

Mahsoub S, Hasserjian R, Chen W, Gagan J, Madanat Y, Weinberg O. USCAP 2022 Abstracts: Hematopathology (851-976). *Modern Pathology*. 2022;35:1016-1162. doi:[10.1038/s41379-022-01041-7](https://doi.org/10.1038/s41379-022-01041-7).

Zhou Y. USCAP 2022 Abstracts: Hematopathology (851-976). *Modern Pathology*. 2022;35:1016-1162. doi:[10.1038/s41379-022-01041-7](https://doi.org/10.1038/s41379-022-01041-7).

Bernard E, Tuechler H, Greenberg PL, et al. Molecular International Prognostic Scoring System for Myelodysplastic Syndromes. *NEJM Evid*. 2022;1(7):EVIDoa2200008. doi:10.1056/EVIDoa2200008.

Zhao D, Eladl E, Zarif M, et al. Molecular characterization of AML-MRC reveals TP53 mutation as an adverse prognostic factor irrespective of MRC-defining criteria, TP53 allelic state, or TP53 variant allele frequency. *Cancer Med*. 2023;12(6):6511-6522. doi:10.1002/cam4.5421.

Bataller A, Chien KS, Sasaki K, et al. PHF6 mutations in myelodysplastic syndromes, chronic myelomonocytic leukemia and acute myeloid leukemia. *Leuk Res*. 2023;127:107044. doi:10.1016/j.leukres.2023.107044.

Tefferi A, Fathima S, Alsugair AKA, Aperna F, Natu A, Abdelmagid MG, et al. *PHF6* mutations in chronic myelomonocytic leukemia identify a unique subset of patients with distinct phenotype and superior prognosis. Am J Hematol. 2024 Dec;99(12):2321-2327. doi: 10.1002/ajh.27492.

Erdos, Katie, et al. Validating the MPN Personalized Risk Calculator in 475 Patients with Polycythemia Vera. Blood, vol. 144, no. Supplement 1, 2024, pp. 4558–4558, <https://doi.org/10.1182/blood-2024-210632>.

Beas F, Novoa Jáuregui S, Gabarrós-Subirà M, et al. Evaluation of the Prognostic Impact of STAG2 in the Molecular International Prognostic Scoring System for Myelodysplastic Syndromes. *Blood*. 2024;144(Supplement 1):6742. doi:[10.1182/blood-2024-206758](https://doi.org/10.1182/blood-2024-206758)

Perusini MA, Andrews C, Atenafu EG, et al. Real-World Experience with CPX-351 for Secondary Acute Myeloid Leukemia: Comparison with FLAG-IDA in a Propensity Score Matching Analysis. *Blood*. 2024;144(Supplement 1):1506. doi:[10.1182/blood-2024-204004](https://doi.org/10.1182/blood-2024-204004).

Kubota Y, Gu X, Terkawi L, Bodo J, Przychodzen BP, Awada H, et al. Molecular and clinical analyses of *PHF6* mutant myeloid neoplasia provide their pathogenesis and therapeutic targeting. Nat Commun. 2024 Feb 28;15(1):1832. doi: 10.1038/s41467-024-46134-w.

Fathima S, Alsugair A, He R, et al. Myeloid neoplasms with PHF6 mutations: context-dependent genomic and prognostic characterization in 176 informative cases. *Blood Cancer J*. 2025;15(1):28. Published 2025 Mar 1. doi:10.1038/s41408-025-01231-x.

**Table S2. Multivariate analysis 1:** Cox proportional hazards model for overall survival.

| ***Variable*** | ***HR*** | **95% CI** | ***P-value*** |
| --- | --- | --- | --- |
| *PHF6^MUT^* | 1.02 | 1.00 - 1.05 | 0.075 |
| Age at diagnosis | 0.98 | 0.93 - 1.02 | 0.3 |
| Sex |  |  |  |
| Male | — | — |  |
| Female | 1.51 | 0.68 - 3.34 | 0.3 |
| WHO-5 Category |  |  |  |
| AML | — | — |  |
| MDS | 0.41 | 0.15 - 1.14 | 0.088 |
| MDS/MPN (CMML) | 0.23 | 0.07 - 0.76 | **0.016** |
| MPN | 0.03 | 0.00 - 0.22 | **<0.001** |
| Blasts PB | 0.99 | 0.97 - 1.01 | 0.5 |
| Performance status*** |  |  |  |
| ECOG 3 | 2.05 | 0.43 - 9.86 | 0.4 |
| ECOG 2 | 2.46 | 0.74 - 8.25 | 0.14 |
| ECOG 1 | 1.04 | 0.43 - 2.47 | >0.9 |
| CI: Confidence interval; HR: Hazard Ratio; WHO-5: Fifth Classification of the World Health Organization; AML: Acute Myeloid Leukemia; MDS: Myelodiplastic Syndrome; MPN: Chronic Myeloproliferative Neoplasm; MDS/MPN: overlap MDS and MPN; PB: Peripheral Blood; CMML: Chronic Myelomonocytic Leukemia: ECOG: Eastern Cooperative Oncology Group.  * ECOG = 0 is the comparison contrast.  **Table S3. Multivariate analysis 2:** Cox proportional hazards model for overall survival. Including age, sex, hemoglobin, co-mutational profile, and cytogenetic profile.   \| **Variable** \| ***HR*** \| **95% CI** \| ***P-value*** \| \| --- \| --- \| --- \| --- \| \| *PHF6^MUT^* \| 1.03 \| 1.00 - 1.06 \| **0.024** \| \| Age at diagnosis \| 1.00 \| 0.96 - 1.04 \| 0.8 \| \| Sex \|  \|  \|  \| \| Male \| — \| — \|  \| \| Female \| 2.10 \| 0.95 - 4.63 \| 0.066 \| \| Hemoglobin \| 0.96 \| 0.77 - 1.19 \| 0.7 \| \| Cytogenetic \|  \|  \|  \| \| Abnormal Karyotype \| — \| — \|  \| \| Complex Karyotype \| 2.68 \| 0.67 - 10.7 \| 0.2 \| \| *DNMT3A* \| 1.25 \| 0.52 - 3.01 \| 0.6 \| \| *NRAS* \| 1.83 \| 0.68 - 4.91 \| 0.2 \| \| *ASXL1* \| 0.41 \| 0.17 - 1.01 \| 0.052 \| \| *U2AF1* \| 1.41 \| 0.42 - 4.79 \| 0.6 \| \| *TP53* \| 0.53 \| 0.18 - 1.55 \| 0.2 \| \| *RUNX1* \| 1.86 \| 0.82 - 4.19 \| 0.14 \|  \| CI: Confidence interval; HR: Hazard Ratio. \| \| \| \| \| --- \| --- \| --- \| --- \|   **Table S4. Multivariate analysis:** Cox proportional hazards model for time-to-next treatment.   \| **Variable** \| ***HR*** \| **95% CI** \| ***P-value*** \| \| --- \| --- \| --- \| --- \| \| *PHF6^MUT^* \| 0.97 \| 0.91 **-** 1.05 \| 0.5 \| \| Age at diagnosis \| 0.96 \| 0.91 **-** 1.02 \| 0.2 \| \| Sex \|  \|  \|  \| \| Male \| — \| — \|  \| \| Female \| 0.80 \| 0.35 **-** 1.86 \| 0.6 \| \| WHO-5 Category \|  \|  \|  \| \| AML \| — \| — \|  \| \| MDS \| 0.06 \| 0.00 **-** 1.26 \| 0.070 \| \| MDS/MPN \| 0.08 \| 0.00 **-** 1.80 \| 0.11 \| \| MPN \| 0.01 \| 0.00 **-** 0.35 \| **0.009** \| \| Blasts PB \| 1.00 \| 0.96 **-** 1.05 \| >0.9 \| \| Performance status* \|  \|  \|  \| \| ECOG 3 \| 0.00 \| 0.00 **-** 2.37 \| 0.089 \| \| ECOG 2 \| 0.03 \| 0.00 - 0.84 \| **0.039** \| \| ECOG 1 \|  \|  \|  \|   CI: Confidence interval; HR: Hazard Ratio; WHO-5: Fifth Classification of the World Health Organization; AML: Acute Myeloid Leukemia; MDS: Myelodiplastic Syndrome; MPN: Chronic Myeloproliferative Neoplasm; MDS/MPN: overlap MDS and MPN; PB: Peripheral Blood; CMML: Chronic Myelomonocytic Leukemia: ECOG: Eastern Cooperative Oncology Group.  * ECOG = 0 is the comparison contrast.  **Table S5. Multivariate analysis:** Cox proportional hazards model for time-to-next treatment. Including age, sex, hemoglobin, co-mutational profile, and cytogenetic profile.   \| **Variable** \| ***HR*** \| **95% CI** \| ***P-value*** \| \| --- \| --- \| --- \| --- \| \| *PHF6^MUT^* \| 1.02 \| 0.96 - 1.09 \| 0.5 \| \| Age at diagnosis \| 1.03 \| 0.98 - 1.08 \| 0.3 \| \| Sex \|  \|  \|  \| \| Male \| — \| — \|  \| \| Female \| 1.85 \| 0.68 - 5.05 \| 0.2 \| \| Hemoglobin \| 1.09 \| 0.91 - 1.30 \| 0.3 \| \| Cytogenetic \|  \|  \|  \| \| Abnormal Karyotype \| — \| — \|  \| \| Complex Karyotype \| 237 \| 11.5 - 4.89 \| **<0.001** \| \| *DNMT3A* \| 0.89 \| 0.32 - 2.50 \| 0.8 \| \| *NRAS* \| 2.58 \| 0.72 - 9.24 \| 0.15 \| \| *ASXL1* \| 1.99 \| 0.69 - 5.77 \| 0.2 \| \| *U2AF1* \| 1.13 \| 0.33 - 3.87 \| 0.8 \| \| *TP53* \| 0.55 \| 0.09 - 3.28 \| 0.5 \| \| *RUNX1* \| 1.33 \| 0.36 - 4.96 \| 0.7 \|  \| CI: Confidence interval; HR: Hazard Ratio. \| \| \| \| \| --- \| --- \| --- \| --- \|   **Table S6. Multivariate analysis:** Cox proportional hazards model for time-to- blast transformation.   \| **Variable** \| ***HR*** \| **95% CI** \| ***P-value*** \| \| --- \| --- \| --- \| --- \| \| *PHF6^MUT^* \|  \|  \|  \| \| Age at diagnosis \| 0.99 \| 0.73 - 1.34 \| >0.9 \| \| Sex \|  \|  \|  \| \| Male \| — \| — \|  \| \| Female \| 2.26 \| 0.16 - 32.4 \| 0.5 \| \| WHO-5 Category \|  \|  \|  \| \| AML \| — \| — \|  \| \| MDS \| 3.19 \| 0.03 - 355 \| 0.6 \| \| MDS/MPN \| 1.60 \| 0.02 - 114 \| 0.8 \| \| MPN \|  \|  \|  \| \| Blasts PB \| 0.55 \| 0.23 - 1.29 \| 0.2 \| \| Performance status* \|  \|  \|  \| \| ECOG 3 \| 0.11 \| 0.00 - 226 \| 0.6 \| \| ECOG 2 \|  \|  \|  \| \| ECOG 1 \|  \|  \|  \|   CI: Confidence interval; HR: Hazard Ratio; WHO-5: Fifth Classification of the World Health Organization; AML: Acute Myeloid Leukemia; MDS: Myelodiplastic Syndrome; MPN: Chronic Myeloproliferative Neoplasm; MDS/MPN: overlap MDS and MPN; PB: Peripheral Blood; CMML: Chronic Myelomonocytic Leukemia: ECOG: Eastern Cooperative Oncology Group.  * ECOG = 0 is the comparison contrast.  **Table S7. Multivariate analysis:** Cox proportional hazards model for time-to-blast transformation. Including age, sex, hemoglobin, co-mutational profile, and cytogenetic profile.   \| **Variable** \| ***HR*** \| **95% CI** \| ***P-value*** \| \| --- \| --- \| --- \| --- \| \| *PHF6^MUT^** \|  \|  \|  \| \| Age at diagnosis \| 0.90 \| 0.74 - 1.09 \| 0.3 \| \| Sex \|  \|  \|  \| \| Male \| — \| — \|  \| \| Female \| 1.42 \| 0.04 - 46.5 \| 0.8 \| \| Hemoglobin \| 1.48 \| 0.71 - 3.10 \| 0.3 \| \| Cytogenetic \|  \|  \|  \| \| Abnormal Karyotype \| — \| — \|  \| \| Complex Karyotype** \|  \| 0.00 - Inf \| >0.9 \| \| *DNMT3A* \|  \|  \|  \| \| *NRAS* \| 0.07 \| 0.00 - 5.51 \| 0.2 \| \| *ASXL1* \| 0.01 \| 0.00 - 0.50 \| **0.022** \| \| *U2AF1* \|  \|  \|  \| \| *TP53* \| 0.48 \| 0.01 - 3.4 \| 0.7 \| \| *RUNX1* \|  \|  \|  \|  \| CI: Confidence interval; HR: Hazard Ratio.  *The HR cannot be calculated, probably due to the small sample size.  **Extreme HR and CI estimates, probably due to small sample size (separation problem; Heinze G. A comparative investigation of methods for logistic regression with separated or nearly separated data. *Stat Med*. 2006;25(24):4216-4226. doi:10.1002/sim.2687). \| \| \| \| \| --- \| --- \| --- \| --- \|   **Table S8. Multivariate analysis:** Cox proportional hazards model for progression-free survival.   \| **Variable** \| ***HR*** \| **95% CI** \| ***P-value*** \| \| --- \| --- \| --- \| --- \| \| *PHF6^MUT^* \| 1.02 \| 1.00 - 1.05 \| 0.082 \| \| Age at diagnosis \| 0.99 \| 0.96 - 1.03 \| 0.7 \| \| Sex \|  \|  \|  \| \| Male \| — \| — \|  \| \| Female \| 1.15 \| 0.68 - 1.93 \| 0.6 \| \| WHO-5 Category \|  \|  \|  \| \| AML \| — \| — \|  \| \| MDS \| 0.44 \| 0.18 - 1.07 \| 0.071 \| \| MDS/MPN \| 0.36 \| 0.14 - 0.97 \| **0.043** \| \| MPN \| 0.10 \| 0.03 - 0.33 \| **<0.001** \| \| Blasts PB \| 1.00 \| 0.99 - 1.02 \| 0.7 \| \| Performance status* \|  \|  \|  \| \| ECOG 3 \| 2.00 \| 0.44 - 8.97 \| 0.4 \| \| ECOG 2 \| 1.59 \| 0.52 - 4.86 \| 0.4 \| \| ECOG 1 \| 1.13 \| 0.55 - 2.33 \| 0.7 \| \| CI: Confidence interval; HR: Hazard Ratio; WHO-5: Fifth Classification of the World Health Organization; AML: Acute Myeloid Leukemia; MDS: Myelodiplastic Syndrome; MPN: Chronic Myeloproliferative Neoplasm; MDS/MPN: overlap MDS and MPN; PB: Peripheral Blood; CMML: Chronic Myelomonocytic Leukemia: ECOG: Eastern Cooperative Oncology Group.  * ECOG = 0 is the comparison contrast. \| \| \| \| | | | |

**Table S9. Multivariate analysis:** Cox proportional hazards model for progression-free survival. Including age, sex, hemoglobin, co-mutational profile, and cytogenetic profile.

| **Variable** | ***HR*** | **95% CI** | ***P-value*** |
| --- | --- | --- | --- |
| *PHF6^MUT^* | 1.02 | 1.00 - 1.05 | **0.039** |
| Age at diagnosis | 1.02 | 0.99 - 1.04 | 0.2 |
| Sex |  |  |  |
| Male | — | — |  |
| Female | 1.07 | 0.63 - 1.82 | 0.8 |
| Hemoglobin | 0.94 | 0.84 - 1.04 | 0.2 |
| Cytogenetic |  |  |  |
| Abnormal Karyotype | — | — |  |
| Complex Karyotype | 5.17 | 1.53 - 17.5 | **0.008** |
| *DNMT3A* | 1.20 | 0.61 - 2.37 | 0.6 |
| *NRAS* | 2.15 | 0.97 - 4.77 | 0.059 |
| *ASXL1* | 0.76 | 0.44 - 1.33 | 0.3 |
| *U2AF1* | 1.30 | 0.59 - 2.88 | 0.5 |
| *TP53* | 0.47 | 0.19 - 1.15 | 0.10 |
| *RUNX1* | 1.13 | 0.60 - 2.12 | 0.7 |

CI: Confidence interval; HR: Hazard Ratio.

**Figures.**

**Figure S1. Cases with mutated *PHF6* and its co-mutations.**

**
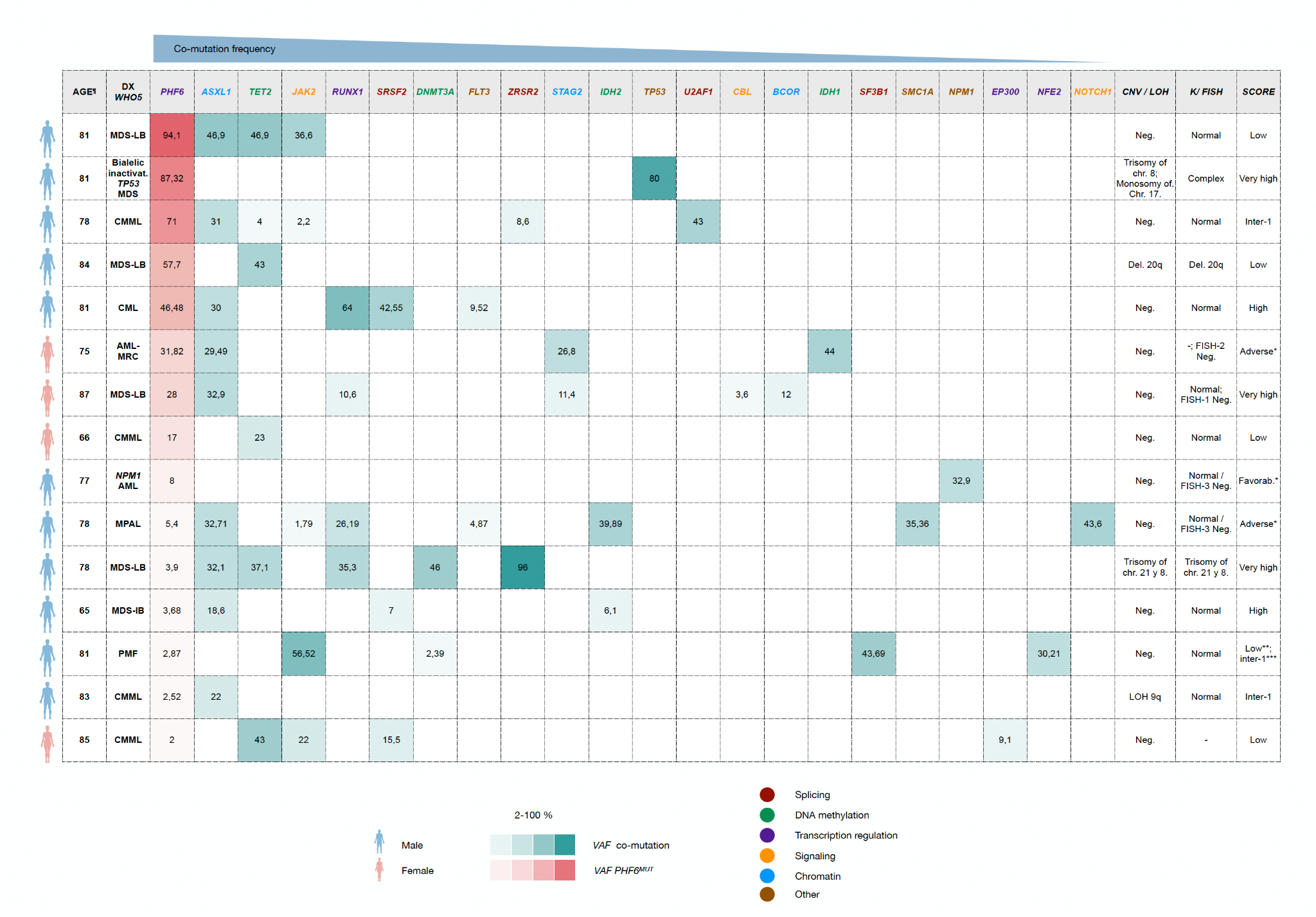
**

¶ Age in years.

DX WHO5: Diagnosis by the 5th edition of the World Health Organization Classification of Haematolymphoid Tumours (Myeloid and Histiocytic/Dendritic Neoplasms); MDS-LB:: Myelodisplastic Syndrome with low blasts; Inactiv.: Inactivation; Chr.: chromosome; CMML: Chronic Myelomonocytic Leukemia; CML: Chronic Myeloid Leukemia; AML-MCR: Acute Myeloid Leukemia - Myelodysplastic Changes Related; MPAL: Mixed-Phenotype Acute Leukemia; MDS-IB: Myelodisplastic Syndrome with increased blasts; PMF: Primary Myelofibrosis; LOH: Loss of Heterozygosity; CNV: Copy Number Variations; K: Karyotype; FISH: Fluorescence in situ hybridization; del.: Deletion; FISH-1: 5q, 7q; FISH-2: 5q, 7q, MLL, t(3;3); FISH-3: MLL, t(3;3). Neg.: negative; Favorab.: favorable; Inter: intermediate risk; *SCORE:* refers to the validated and corresponding risk scale for myeloid neoplasms (IPSS-Mol: MDS; CPSS-Mol: CMML; Sokal index: CML; ELN2022: AML;; DIPSS plus and MIPSS70 v2.0: PMF); * For ELN-2022, not for ELN 2024 FRAIL.. **By DIPSS plus. *** By MIPSS70 v2.0.

**Figure S2. Corrplot matrix of *PHF6* mutation and co-mutations in all myeloid neoplasms.**


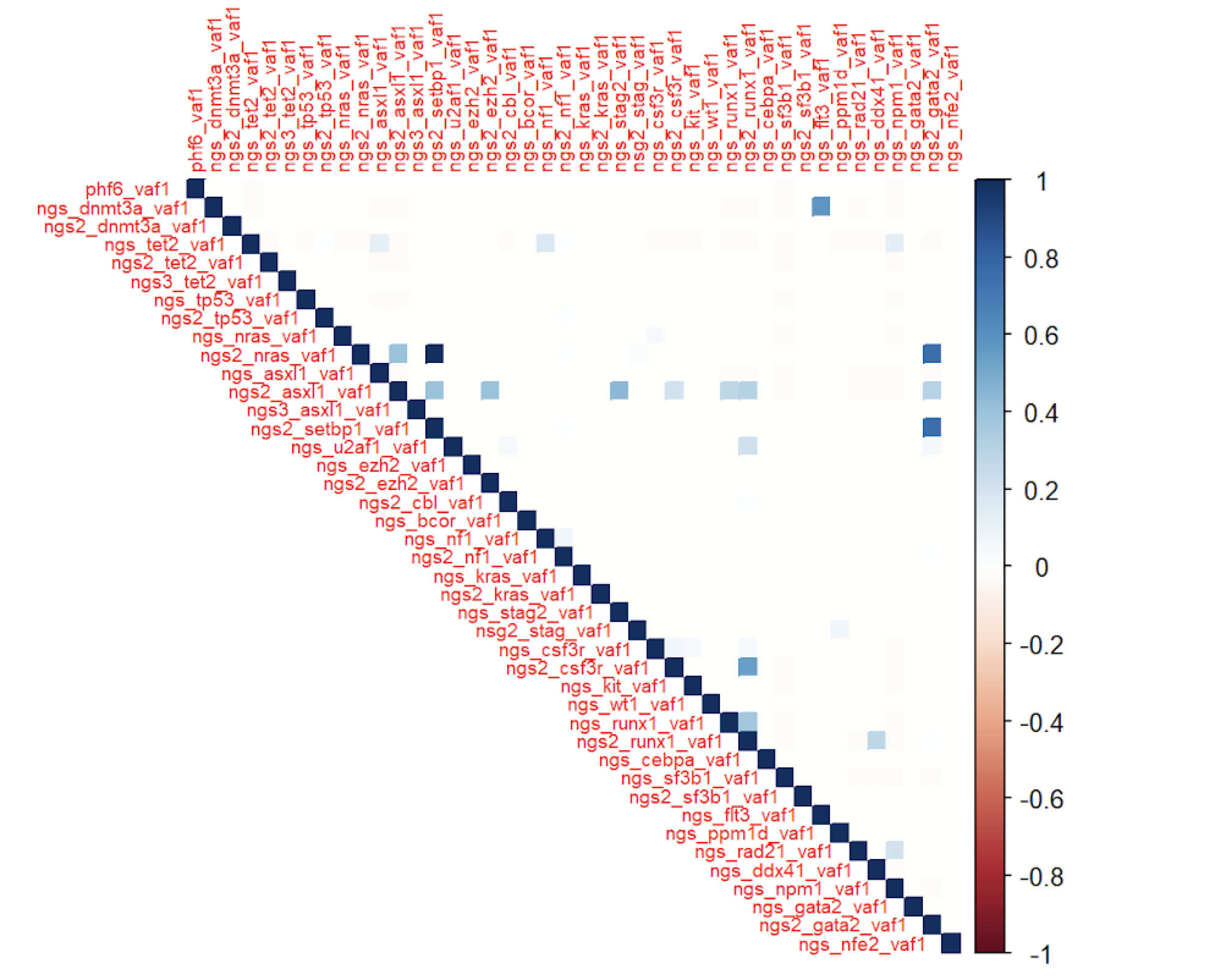


NGS: next-generation sequencing; NGS1, NGS2, NGS3: denotes sequencing at different timepoints; VAF1: variant allele frequency.

**Figure S3.** **Corrplot matrix of *PHF6* mutation and co-mutations in each subgroup of myeloid neoplasm.**

**
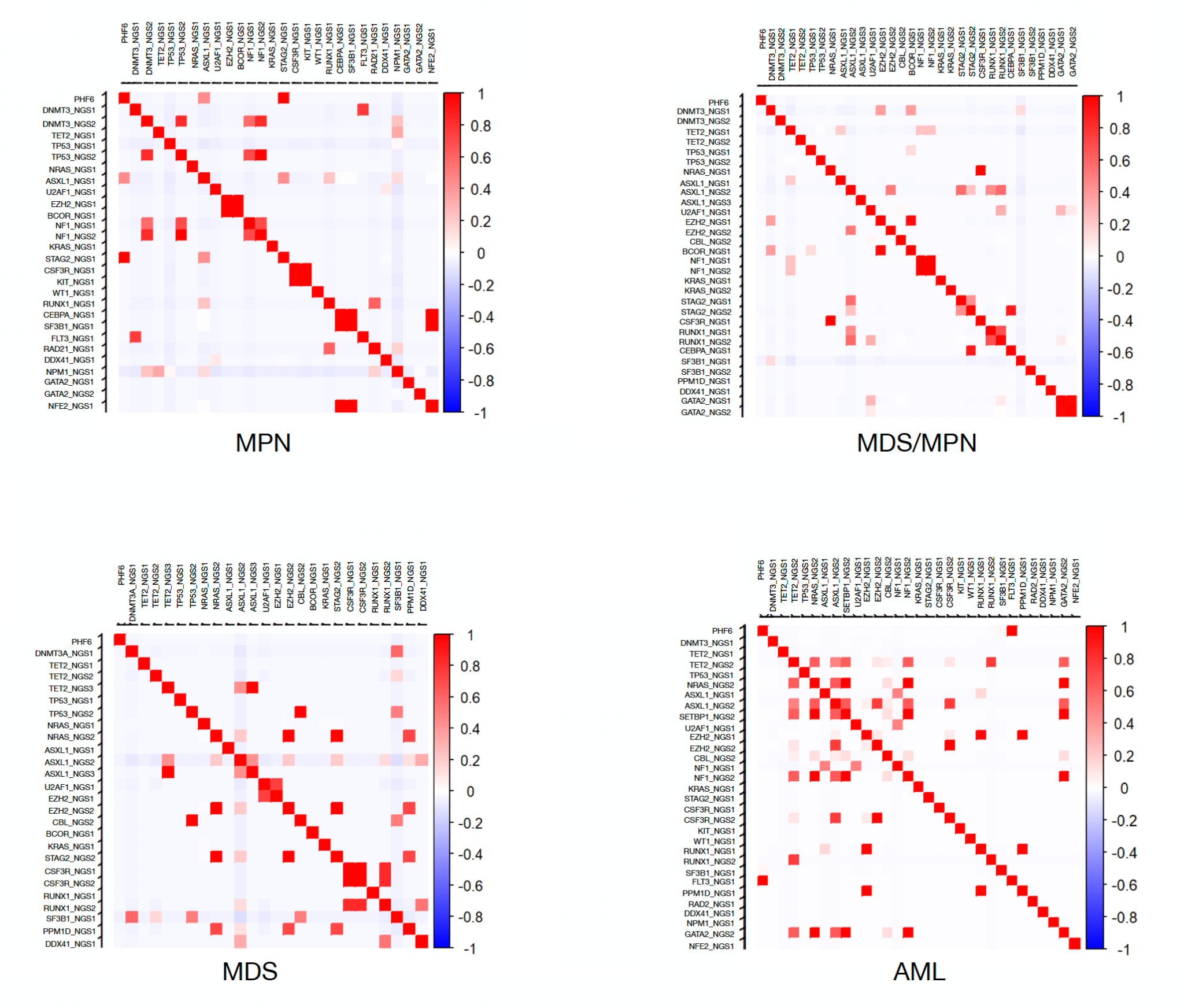
**

MPN: Chronic Myeloproliferative Neoplasm; MDS: Myelodysplastic Syndrome; MDS/MPN: overlap MDS and MPN; AML: Acute Myeloid Leukemia; NGS1, NGS2, NGS3: denotes sequencing at different timepoints.

**Figure S4. *Random Forest Model:* importance of each mutation and its variant allelic frequency in mortality.**


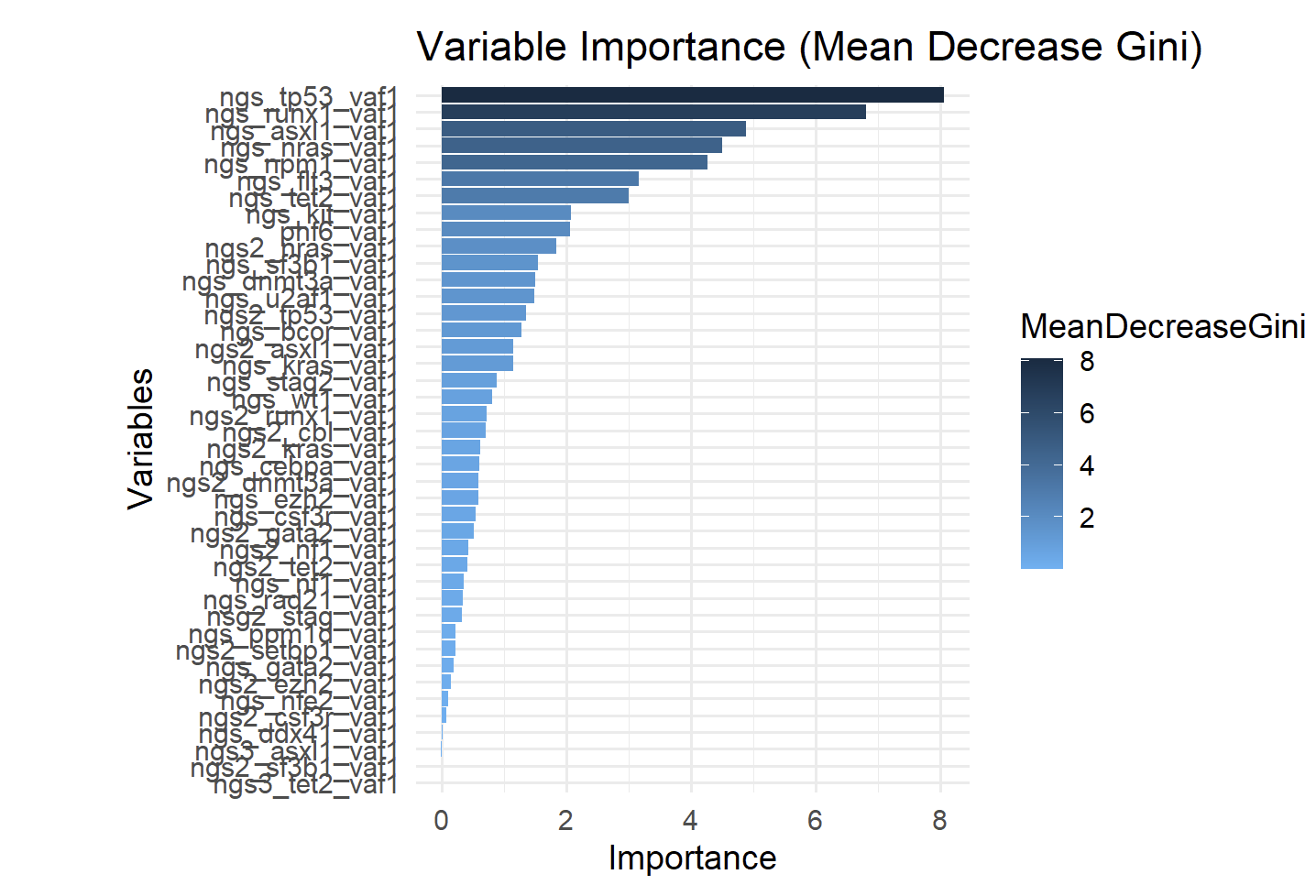


NGS: next-generation sequencing; NGS1, NGS2, NGS3: denotes sequencing at different timepoints; VAF1: variant allele frequency.

* Gini metric: measure of total variance; measure of purity of the “node” (bifurcation); see methodology, statistics section.

**Figure S5.** **Overall survival according to *PHF6* gene status**

***
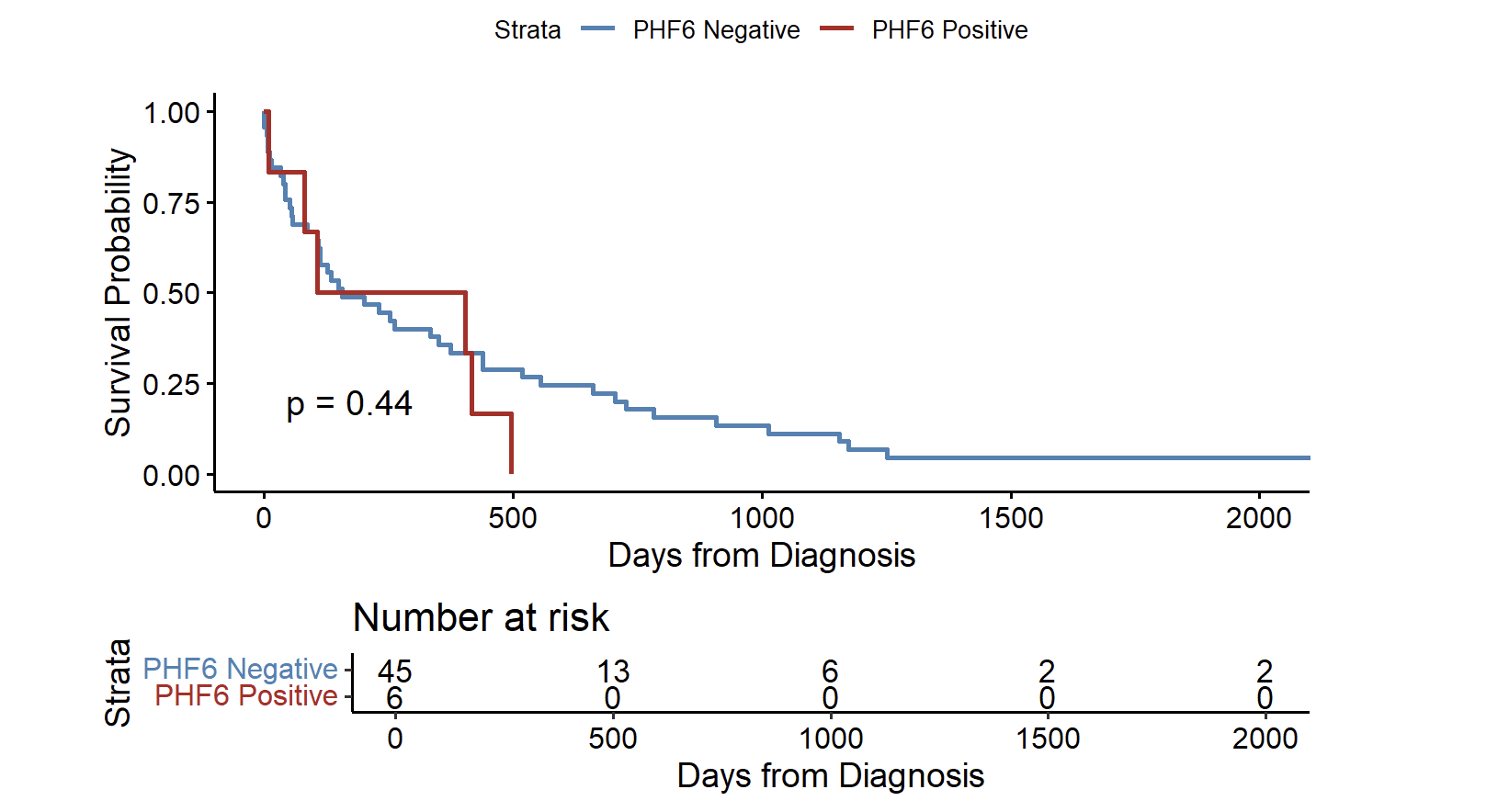
***

**Figure S6. Progression-free survival according to *PHF6* gene status**


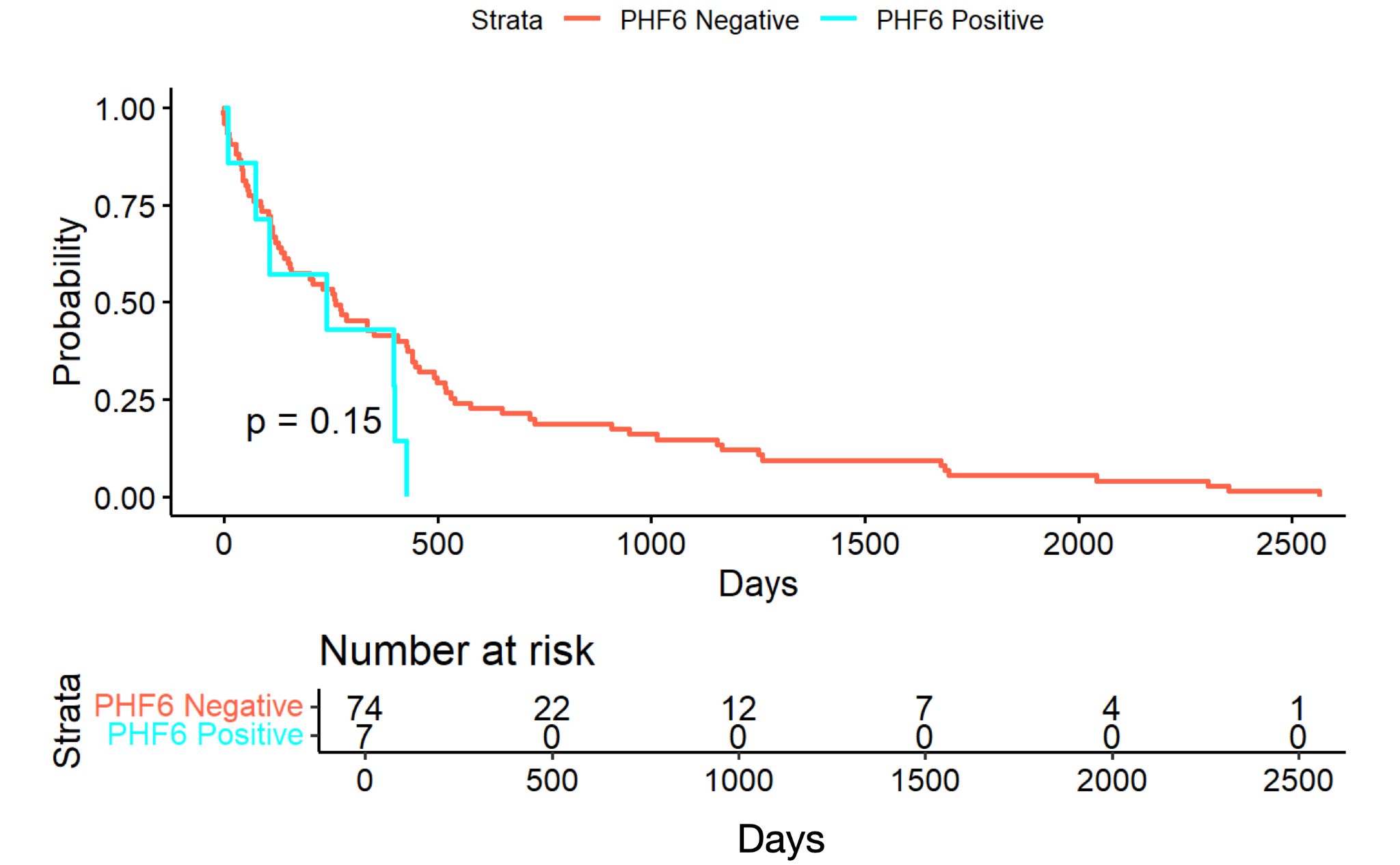


**Figure S7. PRISMA.**
















*From:*  Page MJ, McKenzie JE, Bossuyt PM, Boutron I, Hoffmann TC, Mulrow CD, et al. The PRISMA 2020 statement: an updated guideline for reporting systematic reviews. BMJ 2021;372: n71.  <https://doi.org/10.1136/bmj.n71>
